# Health Information System Role-Based Access Control Current Security Trends and Challenges

**DOI:** 10.1155/2018/6510249

**Published:** 2018-02-19

**Authors:** Marcelo Antonio de Carvalho Junior, Paulo Bandiera-Paiva

**Affiliations:** Health Informatics Department, Federal University of Sao Paulo, Sao Paulo, SP, Brazil

## Abstract

**Objective:**

This article objective is to highlight implementation characteristics, concerns, or limitations over role-based access control (RBAC) use on health information system (HIS) using industry-focused literature review of current publishing for that purpose. Based on the findings, assessment for indication of RBAC is obsolete considering HIS authorization control needs.

**Method:**

We have selected articles related to our investigation theme “RBAC trends and limitations” in 4 different sources related to health informatics or to the engineering technical field. To do so, we have applied the following search query string: “Role-Based Access Control” OR “RBAC” AND “Health information System” OR “EHR” AND “Trends” OR “Challenges” OR “Security” OR “Authorization” OR “Attacks” OR “Permission Assignment” OR “Permission Relation” OR “Permission Mapping” OR “Constraint”. We followed PRISMA applicable flow and general methodology used on software engineering for systematic review.

**Results:**

20 articles were selected after applying inclusion and exclusion criteria resulting contributions from 10 different countries. 17 articles advocate RBAC adaptations. The main security trends and limitations mapped were related to emergency access, grant delegation, and interdomain access control.

**Conclusion:**

Several publishing proposed RBAC adaptations and enhancements in order to cope current HIS use characteristics. Most of the existent RBAC studies are not related to health informatics industry though. There is no clear indication of RBAC obsolescence for HIS use.

## 1. Introduction and Background

Access control is a paramount feature of any secured system. Generally speaking, it provides for subject-to-object segregation according to a security policy implementation at a given system. It can be divided in three phases in which the initial two are related to subject interaction and the third to the object: identification, authentication, and authorization.

Users who want access to a system are prompted by identification processes that may vary from simple username request to digital certificates or biometrics interaction on more robust environment. The main intention is to collect a unique ID (univocal and unambiguous ID), so actions related to that user during security audit can provide for user's accountability. Authentication methods are used to prove the user's identity allegation on subsequent phase. The intention here is to bind some authentication factor that only a legitimate user is supposedly able to provide something he knows, something he has, or something he is. Only then, at the third and final phases, the authorization takes place and the list of controlled objects is defined in conjunction with the permissions granted to the user. The intention at this point is to allow only actions that match/dominate user's (subject) permissions on each and every object (system function or information).

Role-based access control (RBAC) addresses the needs for authorization control over objects and builds up adding the maintenance/administration feature of grouping users that have the same permissions/needs into roles. Users can hence be made members of a certain role according to their responsibilities or corporate position and can be later be reassigned to another role without impacting the underlying access control infrastructure [1, 2]. From an administrative point of view, this scheme allows the user to keep discretionary capability over his own objects but also permits a system administrator to dictate corporate rules (security policy) that affect the user. The ability to group people in such a way may lead also to achieving other security properties such as least privilege and separation of duties' best practices. This access granting granularity is appealing to health information systems (HIS) environments where object restrictions may vary according with organization' policy, professional abilities, or functions as well as by explicit information-owner consent for instance.

RBAC was formalized in 1992 and published by the US National Institute of Standards and Technology (NIST) in 2000 [2]. It was later adopted and formally published as an American National Standards Institute (ANSI) standard in 2004 (ANSI INCITS 359-2004 American National Standard for Information Technology—role-based access control). Currently, a newest ANSI version (2012) is available but several discussions and debates are being conducted to assess its actual alignment, considering current trends and new system environments [3–5].

The RBAC model is composed of the core, hierarchical, static separation of duty relations and the dynamic separation of duty relation components and was intended to cope with single-organization security policy strategy [5].

Using core or flat functionality, permissions allocated to roles are bound to user sessions and the authorization decision is made by checking object mapping. This is dynamically performed for each and every protected object resource in the system. In the RBAC concept, there is no need to repeat permissions on different groups. That is because with the hierarchy property, one role can be related to another and associated constrains and permission sets. By the same token, different roles can be assigned to a single user, hence accumulating permissions for those who need a more flexible access control, considering multiple job position or high-ranked staff that needs to incorporate subordinate access capabilities. The stablished relationship chains users to his or her roles and then to his or her permissions.

**Figure 1:**
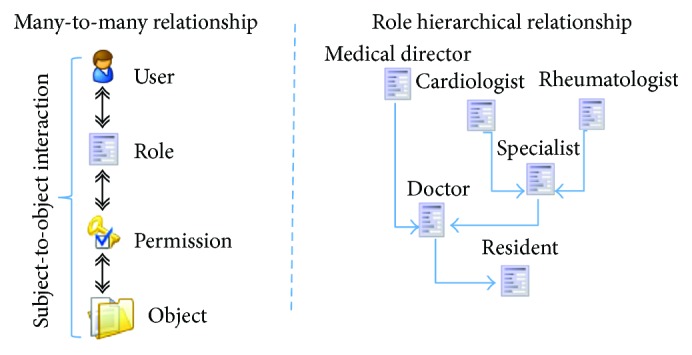
User, roles, and permission relationship and role hierarchy accumulating access permissions over an EHR object representation.

In the illustration (Figure 1), medical director, cardiologists, and rheumatologists share doctor and resident permission set. Cardiologists and rheumatologists share specialists' permission. All three have their own permission set.

The static and dynamic separation of duty components can be used to segment authority among different users to tighten even more the authorization to object in a way a restricted action cannot be performed alone by a single system user.

HIS are an example of environment that needs tight control over its functions and information. The healthcare industry uses RBAC massively on its systems leveraging these properties. The Brazilian Society of Health Informatics (SBIS—http://www.sbis.org.br/) electronic health records (EHR) certification program now checks RBAC characteristics for its systems certification program during audits as a newly added mandatory requirement. This addition is part of a periodic review and requirement updates adopted by SBIS to stay current with EHR needs and industry maturity [6]. Version 3.3- and now 4.2-certified systems both have role capability attestation for access control, considering the different needs of health professionals at health-care provision over system. More specifically, the requirements on NGS1.04—authorization access control—attest for RBAC features.

The ability to comply with health industry needs is uncertain as RBAC may need adaption to new realities as cloud computing, grant delegation, emergency situations, multiple-tenancy environments, and other unpredicted scenarios that challenge the basic RBAC capabilities. Security trends and challenges may refer to healthcare procedural needs reflected in systems as early described, more focus on access control architecture and feature capabilities to meet those gaps, but also may refer to implementation specifics. Common Weakness Enumeration (CWE, https://cwe.mitre.org/) for software weaknesses (IDs: 774, 417, and 225) can be mapped to the general use of RBAC. Common Vulnerabilities and Exposures (CVE, https://cve.mitre.org/) has currently 37 publicly known security vulnerabilities listed related to RBAC use on different systems.

This article discusses authorization issues and RBAC security limitations on HIS based on a literature review. The content for that purpose is divided as follows: Objectives and Methods description, where research theme selection and literature review tools, methods, and rationale are described; Literature Review Classification, where we group and distinguish all found research approaches from selected articles; RBAC Current Trends and Limitations, where the authorization issues to comply to HIS needs is discussed; and Conclusions.

## 2. Objectives and Methods

This article objective is to highlight implementation characteristics, concerns, or limitations over RBAC use on HIS, using current literature review for that purpose. Based on the findings, assessment for indication of RBAC is obsolete considering HIS purposes.

### 2.1. Literature Review Tools, Process, and Rationale

This study performs both a “systematic review” and “synthesis” of focused industry international literature review following general methodology and flow used in software engineering [7, 8] along with the applicable quality reporting guidelines defined by PRISMA [9]. PRISMA is becoming a preferable reporting guideline strategy and is a replacement of QUOROM statement.

**Figure 2:**
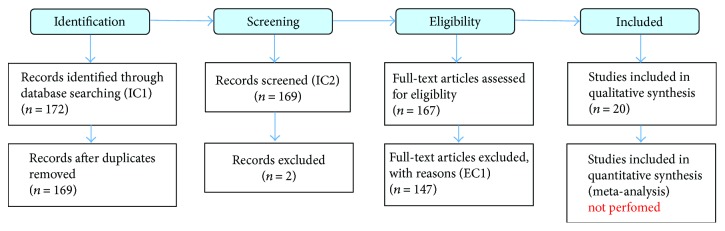
Literature review systematic retrieval process.

More specifically, in this review, we provide for time frame, theme aggregation/synthesis, and mapping of primary studies found discussing RBAC authorization issues. For backward review and interconnection with field study, the existing citations at selected articles were also assessed and can be seen here (http://iccst_link_for_supporting_files/Reference_backward_link). The results from the applied quality checklist can be seen here (http://iccst_link_for_supporting_files/Prisma).  Figure 2 depicts the systematic process adopted. Quality assertion (comparisons) and risk assessment portions of the PRISMA checklist were NOT performed. This was due to the heterogeneous nature/type of included articles. As stated by Zhang et al. and also by Kitchenham and Brereton [7, 10], this proves to be difficult as authors' methodology, exposed data, and approached analysis may be carried out in different fashions and therefore not directly comparable.

That said, and due to the fact that the risk of bias (RoB) using existing quality assertion tools (e.g., Cochrane RoB 2.0 tool and Newcastle-Ottawa Scale (NOS)) is not suitable to assess the data type of study selection in this literature review, reader should be advised that the only assumed quality control is indirectly obtained by the peer review applied on repositories used for content retrieval. As an overall overview of perceived quality indicators on studied material, we can inform that most of the evidence-based software engineering (EBSE) characteristics (level and quality) could be found on texts even though not clearly stated for quality means. These two characteristics refer to study design and conducted method as per author descriptions. The information is more clearly defined on those articles classified as security and efficiency assessments at Table 1.

Based on the intent to find security issues related to RBAC use on HIS systems, we have selected the following research question criteria (RQC) for this study: “what are the security trends and new access-control scenarios that may impact HIS authorization processes using RBAC?” This aims to respond to not only new research directions on future work but also the need to readapt SBIS' authorization requirements on following versions. For that purpose, we have selected IEEExplore, ACM Digital Library, Medline, and Springer as research repositories for study selection on literature review. The document retrieval was based on the following text query (including abstracts when available): “Role-Based Access Control” OR “RBAC” AND “Health information System” OR “EHR” AND “Trends” OR “Challenges” OR “Security” OR “Authorization” OR “Attacks” OR “Permission Assignment” OR “Permission Relation” OR “Permission Mapping” OR “Constraint”. The first and second filters represent the main theme (RBAC), and the field/industry (HIS) we want to check for implications and the subsequent string is part of the areas of interest we want to discuss (that includes RBAC functionalities and potential security issues) including synonymous terms used. The article text portions selected were the title and abstract. This phase was conducted using the JabRef Reference Manager [11] that can perform the filtering and automatic duplicate finding without the actual access to the article file.

### 2.2. Inclusion and Exclusion Criteria

As the first inclusion criteria (IC1), we have selected responses from text query from the research repository. Then, we performed the second filter (IC2), selecting English-written articles only. At the following phase, we wanted to make sure that the papers were related to the HIS access control authorization phase only and the main discussion was RBAC security trends and challenges according to RQC. To achieve that, these exclusion criteria (EC1) were applied manually by assessing an article's specific text portions. We checked for the introduction and conclusion sections of the found articles. This phase was performed by downloading the selected documents using Coordination of Personnel Development and High-level Graduation Foundation- (CAPES-) free access proxy platform.

We then classified the independently agreed selection into findings based on theme focus and type of conclusions for later discussion.

## 3. Literature Review Classification

A wide range of results can be found when searching for RBAC access control on HIS. More than 13,000 documents are retrieved by the search “Role-Based Access Control” AND “Health information System” on Google. The reason behind that is that RBAC is really popular and widely used within HIS scope. In two recent literature reviews aiming for a wider health informatics scope, Señor et al. [12] published that the most preferred access control is the RBAC. This first publication was made after assessing 21 articles out of 1208 initially selected while searching for access control management in EHR. In the following year, their next publication accounted for 27 articles out of 49 while searching for security and privacy in EHR when reporting the use of RBAC as authorization method [13]. For our specific intentions, though, the query string used not only includes EHR, which is a subset of HIS, but also specifies the RBAC authorization features or our main concern terms for discussion (“Trends,” “Challenges,” “Security,” “Authorization,” and “Attacks”). By selecting articles using our inclusion criteria (IC1 and IC2), we found 167 documents at the 4 repositories.

20 articles resulted after manual assessment considering exclusion criteria (EC1). As seen in Table 1, 2 main types/themes were found in this review for the selected scope. The RBAC novels or adaptation classifications refer to the use of external tools to complement missing or unsecure features of RBAC or to a new implementation scenario (novel) proposition. The rationale and motivation for most of this type of classification articles was to cope today's daily healthcare special needs, not originally mapped by traditional RBAC use.

The RBAC security and efficiency assessment type/theme grouped articles that intended to check or assess the RBAC capabilities against a certain condition or to perform comparisons. This includes model observation, case study, flaw detection, and policy-driven compliances for HIS usage or even simply listing the lack of security features needed.

**Figure 3:**
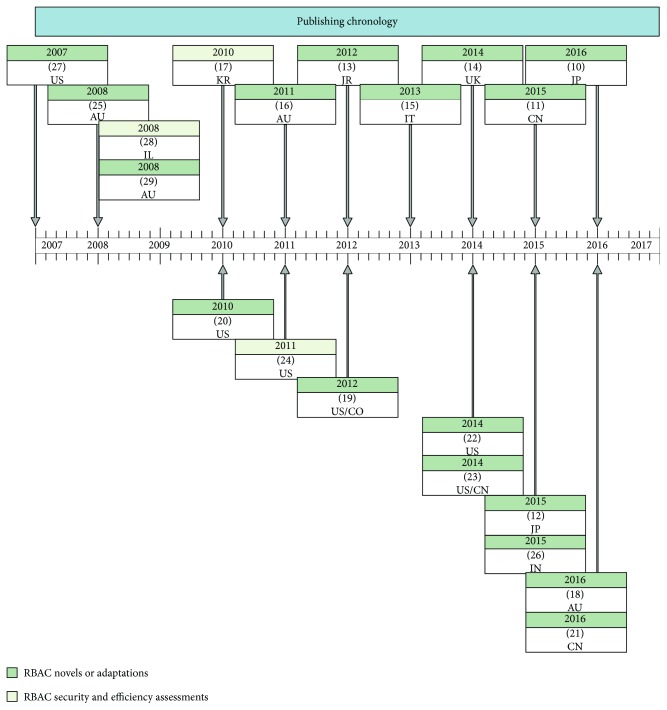
Selected studies' chronological view.

Most of the documents came from IEEExplore and ACM Digital Library. Three article duplicates were removed, and two were scoped out due to language boundaries. As seen in Figure 3, the study period for type/theme is different. They started by performing security assessment mostly and then to propose new approaches and novel implementations to complement RBAC features.

### 3.1. RBAC Novels or Adaptations

Some papers addressed the need for emergency access and/or access delegation features in addition to RBAC. The Khan and Sakamura [14, 16, 17] work describes an RBAC adaptation based on access context. The scenario described is emergency access needed for EHR system information that is currently common on ambulatory but mostly at hospital facilities. The novel proposal accounts for emergency properties to be added to object permission details via a context-policy database addition to the circuit. A delegation token takes this into account to grant access to EHR-protected information under these circumstances. In their second and third work, this token phase is more stressed, advocating the use of eTRON chips (eTRON chips are SIM or USB hardware-type equipped with encryption functionalities) for mutual authentication.

Also, focused on emergency access to protected EHR information over wireless sensor networks (WSN) that implement RBAC, Maw et al. [18] proposed a “breaking the glass” feature allowing access to a previously blocked object under certain user circumstances and obligations.

The delegation and emergency access discussed by these authors are mapped by version 3.3 and 4.2 versions of SBIS' certification requirement set (NGS1.04.07) but are still conditional and a nonmandatory feature.

Other authors included access segmentation or object-detailed representation to propose a granular view for improved authorization decision or interdomain access over RBAC. Liu et al. [15] proposed a novel based on RBAC to be used at nontrusted EHR storage environment (i.e., cloud computing public offers). In this proposition, a trusted key entity is added to the components so hierarchical identity-based encryption can be implement and the EHR consistency status can be audited externally. At the following year, Zhou et al. [24] also proposed an encryption-based solution to achieve hierarchical identity-based broadcast encryption on RBAC and bind the EHR access policy as access key. Using a similar approach but not advising a single key distribution, Warren and Chi [33] proposed ciphertext policy multiauthority (CPMA) and associated use of RBAC permissions to either allow access considering roles (spatial capability) and time (temporal capability) over encrypted EHR on cloud environments. Zhang et al. [25] had previously formalized this spatial time approach as RBTBAC model.

The Chen and Hoang [20] approach is also concerned with the interdomain and cross-border issues related to the use of cloud-based solutions. Similar to [14, 17], they propose a context-based access decision that adds the “Role Roaming” and the “Active Auditing” to the scheme. Also using an information segmentation approach to store EHR securely in the cloud environment, Premarathne et al. [21] suggest the use of steganography-cryptography to index protected information. Also exploring context-based adaptions built upon RBAC, Bhatti et al. [28] proposed encoding disclosure and privacy rules by using a declarative predicate-based syntax in the policy that is XML-based language they call X-GTRBAC. That proposal also supports interdomain collaboration via federation arrangements and specific policies.

Amato et al. [19] propose a semantic-based RBAC system for a more granular access decision using ontologies to represent healthcare access needs, allowing access to sections of EHR information accordingly. It uses Web Ontology Language (OWL) for proposed ontology representation. Similarly, De la Rosa Algarin et al. [22] proposed a role-slice representation thru XML (role-slice diagram) for more granular queries.

Using Security Assertion Markup Language (SAML), Mchumo and Chi [23] perform a simple case-study to demonstrate interdomain use of RBAC considering this adaptation. Alhaqbani and Fidge [29] stated that interdomain/federation using RBAC alone is not feasible and a combination adding mandatory access control (MAC) and discretionary access control (DAC) is needed.

Basant and Kumar [27] demonstrated a possible bottom-up EHR access control method applied to element and atomic data (database tuples) that is based on information feature vector and access classification algorithm (AC2) to be used in conjunction with RBAC.

A few authors proposed additional security layers to RBAC implementation addressing integrity or confidentiality needs on HIS.

Privacy requirements on top of RBAC implementation were proposed by Liu et al. [26] via the open and trusted health information systems (OTHIS) using a user-centric approach to build up authorization over database table and row level. This approach indirectly reflects a new SBIS requirement mapped in the 4.2 newest publishing. The requirement NGS1.12.02 is still recommended only but clearly states the need for patient discretion over its information access.

### 3.2. RBAC Security and Efficiency Assessments

Lee et al. [30] perform RBAC abnormality analysis using principal component analysis (PCA) method for collecting EHR access traffic over the network. Using intrusion detection system (IDS) and similar techniques, they suggest that the misuse detection over the network can be performed by comparing packages that require RBAC authority validity differently placed in a *x*- and *y*-axis when compared to a forged/attack packages.

Helms and Williams [31] assess four different EHR RBAC-aware systems for implementation evaluation. From the 25 criteria selected, 8 were directly related to RBAC security. The study shows that all four systems failed to implement an RBAC component of separation of duties. Two of them did not use the best practice “permission-role review” from symmetric component.

Beimel and Peleg [32] conducted a controlled experiment comparing authorization policies in different scenarios. The contextual role-based access control (context) model and the situation-based access control (SitBAC) model (introduced by the same author in 2008) resulted equally match when simple queries and basic access cases were reproduced. SitBAC though was more efficient while treating complex access decisions involving different roles and contexts altogether.

## 4. RBAC Current Security Trends and Limitations Mapped on HIS

Considering the different healthcare system scenarios and limitations described by the distinguish approaches and per classification applied in this paper, it is possible to correlate different security trends to be addressed on RBAC-aware HIS. Most of the found articles advocate RBAC adaptations. That is an indication that either role-based access control is a long-lived HIS access control selection or that a feasible replacement is not available at the moment. Yet, several improvements on existing RBAC system is advised to cope with diverse current HIS implementations and use scenarios. Although some articles focused only at describing the need of an additional security layer or different access control model combination [26, 29, 31], we can summarize RBAC current adaptation needs as follows:

- Conditional or emergency access and authorization are delegated.
- Context- and situation access-based solutions are advocated to be adapted over original RBAC features [14, 16–18, 32] requiring additional user's obligation.
- Access segmentation and interdomain/federation scenarios are needed.
- Record-dependent or attribute-based encryption using single or multiple trust supplier [15, 21, 24, 33] is suggested. Fine granular authorization for better security policy representation either using semantic-, time-, or spatial-based approaches is advised [20, 25, 27]. Different languages for role and authorization mapping could be used [19, 22, 23, 28].

### 4.1. Next Steps and Future Work

As we experience applying inclusion criteria at the studies' selection phase, the focus on HIS-related papers removed several articles that discuss RBAC security trends and challenges but in a wider approach or implemented in another industry or usage segment. In fact, when we remove the HIS and EHR from our query, it is possible to notice 309% average increase over our preliminary results from the same repositories. By reading the first articles from this broader query, we can infer that this theme is recurrent in other industries, and therefore, it is reasonable to assume that security issues that also impact HIS are being discussed by other field's researchers and therefore must be incorporated for a more in-depth RBAC assessment on future work.

## 5. Conclusions

50 authors are responsible for the published researches that matched this field of interest. Contributions are related to 10 different countries. Less than one-fourth of the RBAC studies related to our criteria were HIS or EHR related, which indicates most of the researches are focused on other industry or non-industry-related works. As per the publishing dates of found articles, we can see that the topic is relatively recently explored by researchers. 17 articles that advocate RBAC adaptations mostly focus on the following: emergency access, authorization delegation, and interdomain topics. Despite many RBAC limitations and tradeoffs, judging by the number of articles suggesting RBAC adaptations, there is currently no author suggesting full replacement of RBAC for HIS environment. There is no clear indication of RBAC obsolescence for HIS use.

## Figures and Tables

**Table 1:** Content/type of classification for fetched articles.

Type/theme	Selected articles for review	
	Titles	Author(s)
RBAC novels or adaptations	[14–29]	Khan and Sakamura; Liu et al.; Maw et al.; Amato et al.; Chen and Hoang; Premarathne et al.; De la Rosa Algarin et al.; Mchumo and Chi; Zhou et al.; Warren and Chi; Zhang et al.; Liu et al.; Basant and Kumar; Bhatti et al.; Alhaqbani and Fidge
RBAC security and efficiency assessments	[30–32]	Lee et al.; Helms and Williams; Beimel and Peleg
